# Recent Advances of Natural Pentacyclic Triterpenoids as Bioactive Delivery System for Synergetic Biological Applications

**DOI:** 10.3390/foods13142226

**Published:** 2024-07-16

**Authors:** Wendi Teng, Zixiao Zhou, Jinxuan Cao, Qing Guo

**Affiliations:** 1 State Key Laboratory of Food Nutrition and Safety, School of Food Science and Technology, Tianjin University of Science and Technology, Tianjin 300457, China; 2 Key Laboratory of Geriatric Nutrition and Health, School of Food and Health, Beijing Technology and Business University, Beijing 100048, China; wenditeng@btbu.edu.cn (W.T.); zzxiao525@hotmail.com (Z.Z.); caojinxuan@btbu.edu.cn (J.C.)

**Keywords:** pentacyclic triterpenoids, natural small molecules, biological activity, self-assembly, bioactive nanocarriers

## Abstract

Bioactive compounds have drawn much attention according to their various health benefits. However, poor dissolvability, low stability and limited bioavailability largely narrow their applications. Although a variety of nontoxic ingredients have been rapidly developed as vehicles to deliver bioactive compounds in the last few years, most of them are non-bioactive. Pentacyclic triterpenoids, owing to their unique self-assembly and co-assembly behaviors and different physiological functions, can construct bioactive carriers due to their higher biodegradability, biocompatibility and lower toxicity. In this paper, the basic classification, biological activities and physicochemical properties of pentacyclic triterpenoids were summarized. Additionally, applications of self-assembled and co-assembled pentacyclic triterpenoids as bioactive delivery systems to load bioactive components and future research directions were discussed. This study emphasizes the potential of pentacyclic triterpenoids as bioactive delivery systems, offering a new perspective for constructing self- or co-assemblies for further synergetic biological applications.

## 1. Introduction

Natural active components attract much attention due to their bioactivities in enhancing the nutritional values and preventing the risks of various diseases like cancers, obesity and diabetes [1,2]. However, bioactive compounds often cannot be completely absorbed by human body [3]. Most of them are not dissolved in water and are very sensitive to environmental stimuli like heat and oxygen, resulting in the decline of stability and limited bioavailability [4]. Moreover, after the bioactive components passed through the gastrointestinal tract, various enzymes and gastric acids would largely decrease the bioactivity [5]. For example, poor solubility of hydrophobic compounds such as resveratrol greatly prevent their dissolution in water and absorption under gastrointestinal conditions in the human body [6,7]. Liposoluble vitamins are often not stable at the stage of processing and storage, or under gastrointestinal conditions [8]. Meanwhile, limited diffusion of the components crossing the intestinal mucus and low permeability through intestinal epithelium largely impeded their bioavailability [9].

In order to overcome these shortcomings, some food matrices were used to embed bioactive compounds to increase their stability and bioavailability [10,11]. Many scientists are focusing on developing delivery platforms including emulsions, nanoparticles (NPs), liposomes and hydrogels, which increase their water solubility, protect from hostile external conditions, and improve intestinal cell permeability [12,13,14]. Nevertheless, these delivery platforms are principally macromolecules or amphiphilic compounds, showing insufficient bioactivities along with easy aggregation, low encapsulation efficiency (EE), loading capacity (LC) and potential safety concerns [15,16,17]. Thus, it is essential to develop new delivery carriers, especially with good bioactivities or biological activities. Recently, some natural small molecules (NSMs) have shown their potential to function as vehicles for their self-assembled supramolecular nanostructures [18,19,20]. The NSMs with bioactivities exhibited superb effects for bioactive delivery and remarkable synergistic bioactivities [21,22]. Among those NSMs, pentacyclic triterpenoids, as a kind of phytochemical, are secondary plant metabolites with chemical structures consisting of five-membered carbon rings [23]. Pentacyclic triterpenoids attracted great attention owing to their remarkable bioactivities but without toxicity [24]. Additionally, avoiding complicated chemical modification, they could form nanostructures via self-assembly or co-assembly, which would greatly enhance their water solubility, bioactivity and oral bioavailability, with no extra components involved [20]. Further, the self/co-assembled nanostructures have great potential as carriers to embed hydrophobic foods and drugs [4]. Pentacyclic triterpenoids showed synergistic biological activities compared with conventional drug delivery systems. Besides the advantages of higher water solubility, improved stability and controlled release, pentacyclic triterpenoids are capable of targeted delivery, offering promising roles for them except for purely biologically active components. 

Many reviews have been published on the bioactivities or physicochemical properties of pentacyclic triterpenoids [25,26,27]. However, there are no comprehensive reports on the applications of pentacyclic triterpenoids as nanocarriers for the encapsulation of bioactive components. In this review, the basic classification, biological activities and physicochemical properties of pentacyclic triterpenoids were first summarized. Additionally, applications of self-assembled and co-assembled pentacyclic triterpenoids as delivery systems to load bioactive components were compared and discussed. Last, challenges and future research directions of pentacyclic triterpenoids as delivery systems were proposed and analyzed. This work offers comprehensive knowledge of pentacyclic triterpenoids and highlights their potential as bioactive nanocarriers, offering a novel perspective for constructing pentacyclic triterpenoids-based self/co-assemblies for synergetic effects. 

## 2. Distribution of Pentacyclic Triterpenoids in Nature

**Figure 1:**
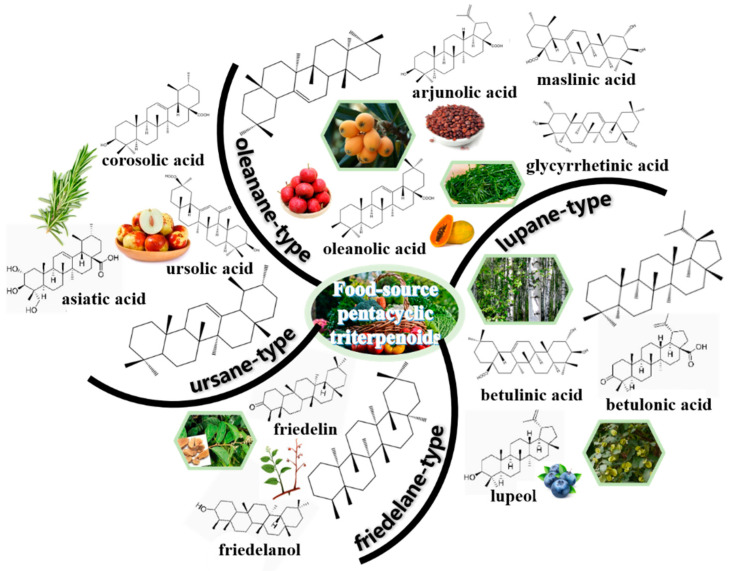
Overview of food-source pentacyclic triterpenoids. Four vital types are summarized, including oleanane-type (e.g., oleanolic acid, glycyrrhetinic acid, arjunolic acid, maslinic acid), ursane-type (e.g., ursolic acid, corosolic acid, asiatic acid), lupane-type (e.g., betulinic acid, betulonic acid, lupeol) and friedelane-type (e.g., friedelin, friedelanol).

Triterpenes are composed of six isoprene units with 30 carbon atoms, are secondary metabolites in nature [23]. Pentacyclic triterpenoids consist of 6-6-6-6-5 or 6-6-6-6-6 as skeletal units, which are one kind of active triterpenes commonly found in plants, monocotyledons, dicotyledons, fungi, marine organisms and pteridophytes [25]. They often exist in Leguminosae, Araliaceae, Cucurbitaceae, Compositae, Poaceae and other plants [28]. According to the skeletal structures, the main types of pentacyclic triterpenes were summarized in Figure 1, including oleanane-type, ursane-type, lupane-type and friedelane-type [29]. 

Oleanane-type are also called β-amyrin-derived pentacyclic triterpenoids. They are always presented in the form of free or combined saponins [30]. Oleanolic acid, glycyrrhetinic acid, arjunolic acid, maslinic acid, esculentoside and polygalacic acid belong to oleanane-type pentacyclic triterpenoids [31]. Ursane-type are as well known as α-amyrin-derived pentacyclic triterpenoids, which have 6-6-6-6-6 as skeletal units. They are often presented in the free form or glycosides from Ilex chinensis, Sanguisorba officinalis and others. Ursolic acid, corosolic acid and asiatic acid are the typical representatives [32]. There are 6-6-6-6-5 as skeletal units for the lupane-type pentacyclic triterpenoids. Among them, betulinic acid, betulonic acid, betulinol and lupeol are widely studied [33]. Friedelane originates from oleanane via methyl translocation. Friedelin and friedelanol, extracted from tripterygium wilfordii, belong to friedelane-type pentacyclic triterpenoids [34]. Furthermore, tingenone exists in Euonymus, and pristimerin is presented in Celastrus [35]. 

Furthermore, a few other types of pentacyclic triterpenoids, like fernane and hopane, could also be found in nature [25]. Fernane and isofernane are the isomers of lupane. Fernenol, mainly extracted from Citrullus colocynthis leaves, is the typical representative of fernane-type pentacyclic triterpenoids [36]. Cylindrin, originating from Imperata cylindrica, is the typical representative of the isofernane type [37]. Hopane is the isomer of fernane. Hopane-type pentacyclic triterpenoids could be extracted from Aquilaria sinensis [38]. 2-hydroxydiplopterol was isolated from the metabolites produced by the halotolerant fungal strain Aspergillus variecolor B-17 [39].

## 3. Biological Activities of Pentacyclic Triterpenoids

**Figure 2:**
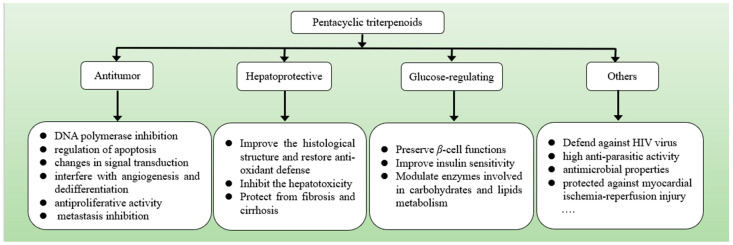
The main biological activities of pentacyclic triterpenoids include anti-tumor, hepatoprotective, glucose regulating and others.

Regularly intaking plant-based foods, such as pawpaw, Chinese ziziphus jujuba and strawberries could lower the incidence of various diseases [40]. The diverse structures of pentacyclic triterpenes determine their varied bioactivities. Pentacyclic triterpenes, with a perceptible improvement in the pharmacological activity, have been shown to have significant therapeutic and tissue-protective effects, including anti-tumor, hepatoprotective, glucose-regulating effects, among others (Figure 2), which are important for the improvement of body functions [25].

### 3.1. Anti-Tumor Activity

In recent research, pentacyclic triterpenoids derived from plants have shown great potential antitumor activity [41]. They could function as molecular targets and cell proliferation regulatory, including inhibiting DNA polymerase, regulating apoptosis, modulating autophagy-related protein, altering the cell cycle in its early phases, changing signal transduction, interfering with angiogenesis and differentiation, inhibiting proliferation and metastasis [42]. Oleanolic acid showed potential antitumor activity in breast cancer, lung cancer and liver cancer, as well as no side effects [43]. The involved pathways included activation of AMP-activated protein kinase pathway, inhibition of PI3K Akt/mTOR/NF-κB signaling pathway, upregulation of p53 mediated activation of mitochondrial apoptotic pathway, and blocking the release of anti-apoptotic inhibitor of apoptotic protein (IAP) family proteins [44]. Ursolic acid exerted anticancer efficacy by inhibiting proliferation, increasing apoptosis, modulating growth receptors, decreasing cellular oxygen consumption, and enhancing NO levels [45]. Betulinic acid demonstrated a selective antitumor activity on human melanoma cell lines both in vitro and in vivo, but minimal toxicity against normal cells was observed [46]. Pentacyclic triterpenoids, naturally isolated active ingredients, could be used as a safer alternative in preventing cancers due to their cytotoxicity to cancer cells but less toxicity to normal cells.

### 3.2. Hepatoprotective Activity

The hepatoprotective activity of pentacyclic triterpenoids has a long history and mature application in China, from the hepatic protection of licorice in the traditional Chinese “Shen Nong Ben Cao Jing” to modern application of over-the-counter hepatoprotective drugs [47,48]. Glycyrrhetinic acid, as one of the most important functional components in licorice, was proven to have a hepatoprotective effect. Glycyrrhetinic acid demonstrated anti-inflammatory effects on propionibacterium acne-induced liver injury by inhibiting macrophage inflammatory protein (MIP)-1 in Kupffer cells by downregulating MyD88 and NF-κB. Glycyrrhetinic acid also regulated the proliferation of liver-infiltrating CD4+T cells and suppressed proinflammatory cytokines [49]. Oleanolic acid was used as a hepatic drug for more than 20 years due to hepatoprotective effects. Researchers concluded oleanolic acid could increase the activity of alanine, reduce liver inflammation, promote the regeneration of liver cells and enable rapid recovery of necrotic liver tissue. It not only prevented liver fibrosis and cirrhosis, but also resisted liver damage caused by various chemicals or active substances [50]. Ursolic acid was shown to protect tetrachloride-induced oxidative damage and greatly increase the survival rate of hepatocytes when compared to tetrachloride-exposed cells, suggesting ursolic acid had strong hepatic protective activity by maintaining the glutathione as well as restraining the malondialdehyde formation according to its radical scavenging functionalities [51]. Additionally, ursolic acid has been shown to protect against ethanol-mediated chronic liver injury through an antioxidant pathway [52]. 

### 3.3. Glucose-Regulating Activity

The antidiabetic activities of pentacyclic triterpenoids have been explored both in vivo and in vitro. Pentacyclic triterpenoids decreased plasma glucose, HbA1c, enhanced plasma insulin, muscle and liver glycogen, and showed protective effects on the liver, eye and heart of diabetic rats, suggesting that they had the potential to prevent diabetes and associated complications [53]. Oleanolic acid alleviates diabetes and metabolic syndrome by improving insulin sensitivity [54]. In rodent studies, oleanolic acid also attenuated fructose-induced hyperglycemia by regulating enzymes involved in carbohydrate digestion, insulin secretion and insulin signaling [55]. Ursolic acid was reported to inhibit pancreatic α-amylase activity and decrease the blood glucose level both in vivo and in vitro [56,57]. 2.5–10 µM of ursolic acid raised the expression of the transcription factors PPARγ, sterol regulatory factor binding protein 1c (SREBP-1c), fatty acid-synthase and fatty acid binding protein 4 (FABP4) [58]. Betulinic acid could enhance insulin sensitivity, and alleviate the blood glucose, inflammation, dyslipidemia and oxidative stress in high-fructose diet-induced metabolic syndrome by activating PI3K/Akt signal pathways [59]. Betulinic acid was also effective at ameliorating TNF-α-induced insulin resistance by preventing the negative regulator of insulin signaling and inflammation-activated protein kinase in adipocytes, thus potentially relieving insulin resistance [29,60].

### 3.4. Others

Pentacyclic triterpenoids have been reported in some other biological activities in the last few years. They were mainly used to inhibit viral replication and reduce reverse transcriptase activity and protease activity as a way to defend against self-replication of HIV [61]. Among 191 pentacyclic triterpenoids reported, 85 have shown high anti-parasitic activity against various species belonging to the genera of plasmodium, leishmania, trypanosoma, as well as various genera of nematoda [62]. Oleanolic acid protects against myocardial ischemia-reperfusion injury by enhancing glutathione and α-tocopherol-mediated mitochondrial antioxidation and ameliorates high glucose-induced endothelial dysfunction by activating PPAR*δ* [63]. Ursolic acid has antimicrobial properties against several harmful pathogens including bacteria, viruses, fungi and parasites [64]. Ursolic acid could kill bacteria and inhibit bacterial growth by altering bacterial cell membranes and adhesion proteins [65]. In addition, cell morphology and expression levels of virulence factors, such as pili and fritillary were regulated by ursolic acid [66]. These highly biological activities with low toxicity made it possible for them to be used as alternatives to traditional chemotherapeutics.

## 4. Self-Assembly and Co-Assembly Properties of Pentacyclic Triterpenoids

Intermolecular noncovalent interactions, including electrostatic interactions, hydrogen bonding, hydrophobic interactions, π-π stacking interactions and van der Waals interactions could drive molecular assembly [67]. Spontaneous self-assembly forming supramolecular architectures have vital potentials in the fields of delivery vehicles, generation of hybrid materials, nanobiodiagnostics, and so on [68]. In the past few years, the self-assembly of natural compounds such as sugars, amino acids and fatty acids has been reported [69]. In 2011, Bag first found that several natural triterpenoids were of nano-meter dimensions with various rigid or flexible lengths, which could self-assemble in different solutions yielding nanoarchitectures like vesicles, fibers, flowers or spheres [70]. Among triterpenoids, pentacyclic triterpenoids are of great significance, which diversified structures lead to self-assembly properties: (1) Their fused 6-6-6-6-6 or 6-6-6-6-5 skeletal units provide a nano-sized rigid backbone; (2) The hydroxyl and carboxyl groups, with the amphiphilic nature and hydrogen bond, provide promising self-assembly properties; (3) The in-built centers of chirality make them useful as chiral building blocks. These different numbers and types of carboxyl, hydroxyl or other groups at different positions and spatial orientations have great influences on the self-assembly properties, leading to various sizes and morphologies [71]. Betulinic acid could self-assemble in 19 organic liquids and alcohol-water mixtures forming gels with a fibrillar network [72]. Betulin and lupeol enabled them to self-assemble in many liquids with various morphologies and similar rheological properties [73]. Likewise, both self-assembled glycyrrhetinic acid and oleanolic acid generated spheres, vesicles or fibrils to form supramolecular gels [74]. Additionally, ursolic acid was shown to stably self-assemble to supramolecular gel enhancing solubility and anticancer effects [75,76].

Co-assembly is an effective and convenient strategy to construct “multi-bioactive” delivery vehicles. Supramolecular co-assembly has been widely used to construct carrier platforms because of their practicality and simplicity [77]. With the increased self-assembled pentacyclic triterpenoids, constructing co-assembled vehicles along with multi-bioactivities has become possible. During the process of co-assembly, NPs could not only maintain the biological activity of each compound but also modulate the self-assembled morphologies and sizes to achieve combination therapy [22].

## 5. Applications of Pentacyclic Triterpenoids as Bioactive Delivery System

### 5.1. Directed Self-Assemblies

Pentacyclic triterpenoids are often widely distributed and could be derived from diversified plants. Their various biological activities help treat or prevent many diseases. However, low physicochemical stability and poor bioavailability limit the applications of bioactive compounds in various fields. For example, the bioavailabilities of curcumin, β-carotene and α-tocopherol were proved to be lower than 5%, which resulted in a waste of cost and time spent to produce them and showed inefficient health-promoting results [78]. In recent decades, encapsulating bioactive compounds to protect from multiple food processing and increase delivery efficiency has attracted great interest from food scientists, but nano-vehicles might go along with high toxicity as well as possible immunogenicity, therefore enhancing product costs and bringing unanticipated risks. Though various nontoxic ingredients have rapidly developed as vehicles to deliver bioactive compounds in the last few years, they are all non-bioactive [79]. Pentacyclic triterpenoids, owing to their unique self-assembly/co-assembly behaviors and different physiological functions, can construct carriers for further synergetic biological applications, which have brought great potential in many fields due to their more biodegradability, biocompatibility and lower toxicity. Until now, only a few pentacyclic triterpenoids have been developed for bioactive delivery, since not all pentacyclic triterpenoids can self-assemble to vehicles with a proper morphology and size, or good biological functions. All self-assembled pentacyclic terpenoids have been explored and compared in how to release active compounds and function as bioactive delivery carriers, and the specific information is listed in Table 1.

#### 5.1.1. Oleanolic Acid

Limited water dispersibility and stability of β-carotene (Car) largely prevent its functions and uses in the field of the food industry. Self-assembled oleanolic acid was used as a delivery system to load hydrophobicCar. After the optimization, LC and EE reached 32.6% and 80.7%, respectively. Self-assembled oleanolic acid NPs could embed up to 300 mg/g of Car. Hydrogen bonding and hydrophobic interactions between oleanolic acid and Car were the main forces promoting self-assembly and capsulation. Compared to free Car, Car/oleanolic acid NPs greatly increased aqueous solubility and protected against UV radiation, ionic strength, and heat. Moreover, Car/oleanolic acid NPs offered delayed release in simulated gastric conditions and controlled release in intestinal conditions. In addition, Car/oleanolic acid NPs showed better hepatoprotective and antioxidant effects [80].

Paclitaxel (PTX) is an anti-cancer chemotherapeutic agent but with certain toxic side effects on normal cells. Self-assembled oleanolic acid was constructed to encapsulate PTX, with 17.1% of the maximal LC and 62.6% of maximal EE [81]. An amount of 280 mg/g of PTX could be encapsulated by oleanolic acid supramolecular NPs. Molecular simulation showed that the assembly process is mainly through hydrophobic interaction and hydrogen bonding interaction. Oleanolic acid could form an assembled nanostructure with the carboxyl group facing the continuous phase and the hydroxyl group dispersed phase. Along with the organic phase volatilized, oleanolic acid molecules gradually aggregate, forming a compactly packed bilayer shell structure through the hydrophobic interaction. Oleanolic acid went on to internally extend loosely to form the intermediate layer of oleanolic acid NPs. When adding the PTX, intermediate layers and the internal hydrophobic area were filled by aggregates via PTX-OA interaction, forming core-shell structures. PTX/oleanolic acid NPs showed slow and sustained release of PTX and were more stable under acidic conditions. In-vitro and in-vivo research demonstrated that PTX/ oleanolic acid NPs had synergistic antitumor effects due to different mechanisms, with an approximately 18% higher rate of tumor inhibition than the PTX group. Liver injury was largely decreased owing to the hepatoprotective activity of oleanolic acid. These studies showed that oleanolic acid could be used as bioactive nanocarriers to deliver hydrophobic nutrients, bioactive components and chemotherapeutic agents, so as to show synergetic effects and lowered side effects.

#### 5.1.2. Ursolic Acid

Self-assembled ursolic acid NPs were developed as a new “self-contained bioactive nanocarrier” to load PTX by hydrogen bonding and hydrophobic interaction. The resulting PTX/ursolic acid NPs showed a uniformly dispersed nanosphere structure with 23.12 ± 1.07% of LC and 94.41 ± 4.28% of EE after optimization. The highest loading capacity of ursolic acid supramolecular NPs for PTX was up to 230 mg/g. They had excellent stability in PBS for at least 15 days, and in serum for 24 h. Likewise, they dissociated gradually and released active components after entering the cells. PTX/ursolic acid NPs significantly prolonged the plasma half-life and showed highly efficient synergistic antitumor efficacy. The inhibition rate in mice was up to 90%, 3.3 times higher than that in the PTX group. Further, PTX/ursolic acid NPs significantly alleviated liver injury, showing that ursolic acid, as a “self-contained bioactive nanocarrier”, was biosafe and enabled reduced liver damage induced by chemotherapeutics via key antioxidant pathways [82].

At the same time, Braja Gopal Bag et al. demonstrated that the vesicular self-assembled ursolic acid gel was capable of entrapping cationic dye Rhodamine B (Rho-B), anionic dye 5,6-carboxfluorescein (CF) and chemotherapeutic agent doxorubicin (DOX) by comparative fluorescence intensity, respectively. Hydrogen bonding involving hydroxyl and carboxyl groups and the dispersion interactions by the triterpenoid backbones might play an important part in the self-assembly. An amount of 87% of Rho B was controllably released after 23 h 30 min at physiological conditions and 75% of DOX was released after 30 min at physiological conditions. Surprisingly, 88% of DOX was released after 60 min at pH 6.6, imitating the low pH environment of hypoxic tumors or endosomes [77]. All these works make ursolic acid possible as a prospective delivery vehicle for scientific and industrial fields.

#### 5.1.3. Betulinic Acid

Glyburide is a promising tested agent for stroke by mediating cerebral edema, but has a limited ability to pass the blood-brain barrier [23]. Betulinic acid NPs were employed as a bioactive multifunctional vehicle to markedly increase the delivery of glyburide to the brain, leading to synergetic effects for ischemic stroke by nuclear factor erythroid 2-related factor 2 (Nrf2)/heme oxygenase (HO-1) antioxidant pathway, compared with either glyburide or betulinic acid NPs. 91% of glyburide was controllably released and retained in the brain over three days [23]. Currently, in clinical practice, a low dose (3 mg/d) of glyburide has been used, thus limiting its efficacy. However, a higher dose of glyburide would lead to hypoglycemia. Betulinic acid NPs decrease glyburide to be exposed to the circulatory system, lowering the risk of hypoglycemia. It not only enhanced the delivery of glyburide to brain tissue, but also decreased the side effects. Furthermore, glyburide-encapsulated betulinic acid NPs showed the simplest and high-efficiency way to intervene in both cerebral edema and oxidation, two critical targets for stroke treatment [51]. Additionally, glyburide/betulinic acid NPs improved patient convenience. Owing to the low brain retention and short blood half-life, continuous infusion for 72 h by osmatic pumps was needed for glyburide administration in vivo [12]. Glyburide/betulinic acid NPs would be sufficient to bring remarkable therapeutic benefits.

#### 5.1.4. Betulonic Acid

Supramolecular self-assembled betulonic acid NPs were formed to load PTX mainly by hydrogen bond and hydrophobic interaction. The contact angle of PTX-loaded betulonic acid NPs was 77.5°, showing improved water dispersibility, and it helped keep stability under acidic conditions. In-vitro and in-vivo data found that PTX/betulonic acid NPs enabled precisive targeting, increased PTX bioavailability by increasing the permeability and retention effect, and showed superb synergistic anti-tumor efficacy by different anti-tumor mechanisms of betulonic acid and PTX. These self-assembled NPs showed various advantages of nanovehicles, like excellent water dispersibility, proper morphologies and sizes, prolonged plasma half-life and enhanced cell uptake and targeting. In addition, it increased biosafety and reduced the toxicity to normal tissues and cells [83].

In photodynamic therapy, chlorin e6 (Ce6) is often used as a photosensitizer because of its efficient antitumor effect. Assembly strategies were used to construct betulonic acid-mediated photosensitizers with no structural modifications. Intermolecular π-π stacking and hydrophobic interactions might be the primary driving forces. Ce6/betulonic acid NPs kept stable in water, cell cultural medium and PBS buffer, and also had better photostability and anti-photobleaching ability than free Ce6. The assembled Ce6/betulonic acid NPs prolonged blood circulation and exhibited a significantly increased, synergistic anticancer efficacy by promoting ROS generation [84].

#### 5.1.5. Arjunolic Acid, Corosolic Acid and Maslinic Acid

Gel could often be utilized as smart scaffolds for extensive delivery, whose fibrillary structures enabled stable release and protected from premature degradation, extending the circulation time and lowering the cytotoxicity. Additionally, the fibrillary networks facilitated to be taken up easily by cells, thus increasing the therapeutic effects. Arjunolic acid, corosolic acid and maslinic acid can also respectively self-assemble to vesicular gels to entrap Rho B, CF or DOX in aqueous solvent mixtures respectively and controlled release of the entrapped molecules at physiological pH or mediated by surfactant, indicating their usefulness as delivery vehicles [85,86,87]. Researchers need to further explore their potential for use in health benefits.

### 5.2. Multi-Components Co-Assemblies

Different from multi-component-directed self-assemblies, supramolecular co-assemblies were novel chemical entities based upon various non-covalent interactions to combine with different components, which could function as co-delivery systems. All co-assembled pentacyclic terpenoids have been explored and the related information is listed in Table 2.

#### 5.2.1. Oleanolic Acid-Glycyrrhetinic Acid/PTX

A pure natural co-assembled delivery system was formed through directly co-assembling oleanolic acid and glycyrrhetinic acid through hydrogen bonding and hydrophobic interactions. PTX could be entrapped in the co-assembled vehicles with 15.1 ± 0.4% of maximum LC and 98.8 ± 1.0% of EE. The maximal loading of glycyrrhetinic acid-oleanolic acid co-assembled NPs for PTX was up to 150 mg/g. PTX-loaded oleanolic acid+ glycyrrhetinic acid NPs were relatively stable under acidic conditions, and also had good dispersion and chemical stability at room temperature over 2 months as well as in serum for at least 48 h. 84 ± 9.9% of PTX could gradually dissociate and continuously released after being engulfed by the cells. In-vitro and in-vivo studies have demonstrated synergistic anticancer activities and the tumor inhibition rate increased from 58.9% to 82.6%. In addition, compared with free PTX, they could alleviate liver damage by regulating key antioxidant pathways and reduce the risk of nanotoxicity, due to the unique anti-inflammatory and hepatoprotective effects of oleanolic acid and glycyrrhetinic acid [22].

#### 5.2.2. Carrier-Free Nanodelivery System

A “core-shell” co-assembly carrier-free system was constructed based on ursolic acid self-assembled as core and epigallocatechin gallate (EGCG) self-polymerized to form a uniform layer as a shell with aptamer modification. The carrier-free system was pH-responsive and kept stable in a normal extracellular environment, but released rapidly in weak acid condition where tumor existed. It increased tumor cellular uptake and long circulation. Ursolic acid combined with EGCG activated the innate immunity resulting in a significant synergistic anti-hepatocellular carcinoma effect. Moreover, the carrier-free system showed low cytotoxicity in normal cells, good biosafety of organic tissues and enlarged tumor accumulation in vivo [90]. Glycyrrhetinic acid/oleanolic acid and Glycyrrhetinic acid/liquidambaric acid NPs were combined via a supramolecular assembly mechanism of non-covalent interactions to form a carrier-free system, which not only enhanced cellular uptake and passive targeting tumor ability, but also arrested cell cycle at different stages of cancer cells and induced apoptosis, demonstrating synergistic anticancer effects with reliable biosafety when comparing to every single component [89]. However, not all the co-assemblies would be effective. For example, co-assembled morphologies were not observed in ursolic acid/betulinic acid, glycyrrhetinic acid/lupeol and ursolic acid/liquidambaric acid [89]. It might be due to hydrophobic forces not driving to form supramolecular nanostructures based on their spatial orientation of carboxyl, hydroxyl or other groups.

## 6. Conclusions and Further Perspectives

Pentacyclic triterpenoids are pivotal natural active components with various biological activities, but poor water dispersibility, poor absorption and rapid metabolism, which is largely influenced their applications. Nowadays, a variety of vehicles have been developed to deliver pentacyclic triterpenoids [91]. Despite these merits, the drawback of these delivery systems is their low loading capacity. For example, the loading capacity of asiatic acid in glyceryltristearate solid lipid nanoparticles is just 2.1% to 5.0% [92]. Merely 3.35% to 12.05% ursolic acid was embedded by a nanostructured lipid carrier coated with chitosan oligosaccharides [93] (Das et al., 2017). Furthermore, large amounts of inactive vehicle materials not only expanded the cost but also brought safety concerns [94].

According to the current research, pentacyclic triterpenoids could be a good matrix that acts as biocompatible nanocarriers. Their spontaneous, reversible self-assembly results from the generation of intermolecular forces, mainly by hydrogen bonds of carboxyl and hydroxyl groups, as well as the dispersing forces of the non-polar triterpenoid skeleton. Discovering the self-assembly properties was recorded as a breakthrough in natural products. Unique nanostructures with various physical characteristics enabled them to form NPs in different sizes and shapes. Surprisingly, the loading capacity of self-assembled/co-assembled pentacyclic triterpenoids was quite higher than that of other delivery systems. Meanwhile, the spontaneous assembly of pentacyclic triterpenoids offered biological activities, which eliminated cytotoxicity and complicated synthesis; it not only provided effective strategies to develop novel therapies, but also helped establish new usage of old drugs.

**Figure 3:**
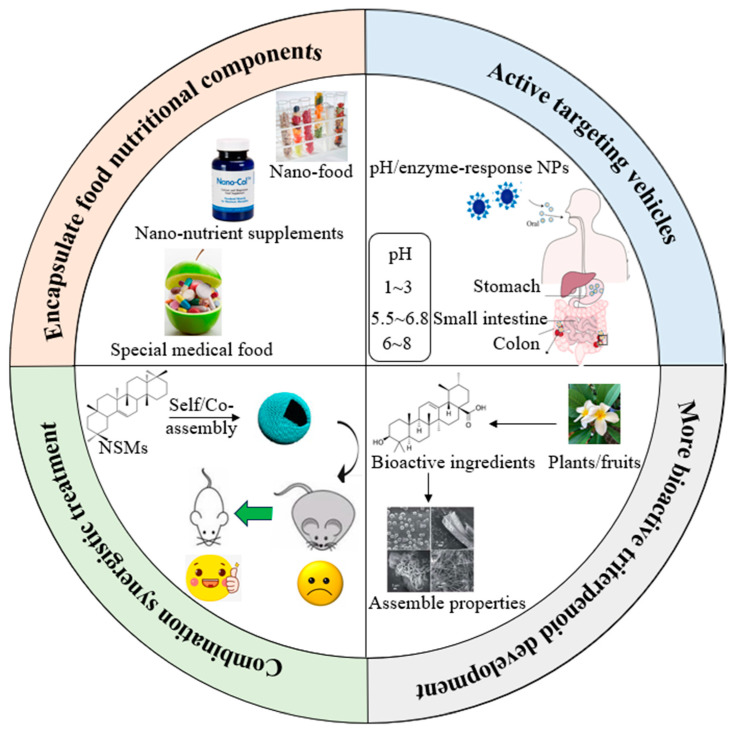
Overview of several research perspectives of pentacyclic triterpenoids in food science [21,76].

Although the self-assembled NSMs have attracted much attention, various theoretical and technological problems still need to be overcome. We have proposed several research perspectives in both basic research and applications (Figure 3): (1) Though assembled pentacyclic triterpenoids were used for drug encapsulation, there have been few studies on entrapping food nutritional components for food applications. Nano-delivery systems based upon pentacyclic triterpenoids can be used in the food industry to develop products such as special medical foods, healthy nano-foods and nano-nutrient supplements to meet special needs for people with limited access to food, digestive and absorption disorders or specific disease states for nutrients or diets. They can also be used to prevent the occurrence of certain health problems and improve health status. It provides a novel strategy for nutraceuticals to improve instability, low bioavailability and synergistic effects with no toxicity. Thus, more in-vivo research is needed to explore the gastrointestinal fate of different pentacyclic triterpenoids as bioactive delivery systems so that their safety and efficacy can be established. The bioavailability assessment of bioactive pentacyclic triterpenoids in various food products still needs to be conducted. In addition, a strict regulatory system managing their applications in the food field would be addressed. (2) There is an increasing focus on exploring precise treatments for the public. Designing “smart” nanoformulations, that can release bioactive compounds from the vehicles with stimulus, is one of the future directions [95]. For example, the chitosan at the surface of nanovehicles facilitated the rapid release of the bioactive compounds to a low-pH environment, a microenvironmental characteristic of the tumors, thus enhancing the therapy efficiency [96]. Pentacyclic triterpenoids-derived NPs endow tissues with targeting ability, allowing for targeted delivery with no additional chemical modifications. The cancer-targeting was reported to be achieved by combining folate, lactoferrin or lactobionic acid residues with the nanocarriers, increasing the cellular uptake [97,98]. Deng et al. reported that betulinic acid could interact with the CB1 receptor, which facilitated efficient brain penetration [23]. Reversible non-covalent interactions could highly respond to stimuli such as pH, light and heat. In addition, the targeting of nanovehicles to the proper tissues or organs could decrease the side effects of treatment. Thus, pentacyclic triterpenoids can be designed as vehicle platforms with stimuli-responsiveness or active targeting to enlarge the anticancer, antimicrobial, antiviral and other biological activities. (3) A large amount of research has reported that self-assembled/co-assembled pentacyclic triterpenoids could enhance their efficiency in antitumor treatment. Surprisingly, cytotoxicity was not observed when incubated with normal cell lines, showing superior selectivity for cancer therapy. Future scientists could not only concentrate on their anticancer effects but also pay more attention to their other biological activities. Owing to the self-assembly and co-assembly properties, it would be possible to develop combined prevention or treatments for metabolic syndrome such as hyperglycemia and hyperlipidemia. A few bioactive pentacyclic triterpenoids could be released in a changing environment, which would also help decrease the dosages, reduce the multi-drug resistance and achieve synergistic therapeutic effects. Further in-vivo and interdisciplinary studies are needed to assess the pros and cons of such bioactive delivery systems over extended periods. All efforts would contribute to the enhancement of natural pentacyclic triterpenoids as a bioactive delivery system for synergetic biological applications, therefore improving the life quality of human beings. (4) Nowadays, self-assembled pentacyclic triterpenoids are almost prepared via conventional or modified emulsion-solvent evaporation method, which limits more pentacyclic triterpenoids with assembled properties and bioactivities to be discovered, considering their wide structural diversities. Larger scales, lack of delivery practicability, low drug loading efficiency and poor aqueous solubility also largely limited their further applications. Thus, developing multiple efficient emerging techniques or modifications to existing methods that could improve scalability, drug loading efficiency, and aqueous solubility, and exploring the physicochemical properties and biological activities of more pentacyclic triterpenoids are urgently needed in the near future, and can significantly help expand the delivery development.

## Figures and Tables

**Table 1:** Nanoparticle carriers for transporting bioactive components.

Carrier	Loaded Compounds	LC&EE	Interaction Forces	Physicochemical Properties	Functional Activities	References
Oleanolic acid	Car	32.6 ± 0.0%; 80.7 ± 0.1%.	Hydrogen bonding; hydrophobic interactions.	Increased aqueous solubility and protection against UV radiation ionic strength, and heat. Delayed release in simulated gastric conditions and controlled release in simulated intestinal conditions.	Enhanced hepatoprotective and antioxidant effects.	[80]
Oleanolic acid	PTX	17.1 ± 0.0%; 62.6 ± 0.0%.	Hydrophobic interactions; hydrogen bonding.	Increased stability, slowed and sustained release under acidic conditions.	Tumor inhibition rate was 76.54 ± 0.66%, about 18% higher than that of PTX group. Reduced liver injury.	[81]
Ursolic acid	PTX	23.12 ± 1.07%; 94.41 ± 4.28%,	Hydrophobic interactions; hydrogen bonding.	More stable in PBS for at least 15 days, in serum for 24 h and also under acidic conditions. Controlled release after entering the cells.	Prolonged plasma half-life. Tumor inhibition rate was 90.2%, 3.3 times higher than that of PTX group. Reduced liver injury.	[82]
Ursolic acid	Rho B/CF/DOX		Hydrogen bonding; Van der Waals interactions.	Controlled release of Rho B at physiological conditions. Slow release of DOX at physiological conditions and pH 6.6.	_	[77]
Betulinic acid	Glyburide	_	_	Increase the delivery to the brain. Controllably released over three days.	Synergetic effects for ischemic stroke by antioxidant and anti-edema.	[23]
Betulonic acid	PTX	_	Hydrogen bonding; hydrophobic interaction.	Improved water solubility. More stable under acidic conditions.	Synergistic anti-tumor efficacy and minimize the side effects.	[83]
Betulonic acid	Ce6	_	π-π stacking; hydrophobic interactions.	Excellent water dispersity. Keep stable in water, cell cultural medium and PBS buffer (pH 7.4). Have better photostability.	prolonged blood circulation. Synergistic anticancer efficacy.	[84]
Arjunolic acid	Rho B/CF/DOX		Hydrogen bonding	Slow release of PTX at physiological pH (7.2).	_	[85,86]
Corosolic acid	Rho B/CF/DOX		Hydrogen bonding; Van der Waals interactions.	Triton X-100-triggered release of Rho-B.	_	[87,88]
Maslinic acid	Rho B/ CF/ DOX		Hydrogen bonding; lipophilic interactions.	Triton X-100-triggered release of DOX.	_	[71]

Car; carotene; PTX: paclitaxel; Rho B: Rhodamine B; CF: 5,6-carboxfluorescein; DOX: doxorubicin; Ce6: chlorin e6.

**Table 2:** Nanoparticle carriers for transporting bioactive components.

Carrier	Loaded Compounds	LC&EE	Interaction forces	Physicochemical Properties	Functional Activities	References
Oleanolic acid -glycyrrhetinic acid	PTX	15.1 ± 0.4%, 98.8 ± 1.0%	Hydrogen bonding; hydrophobic interactions.	Stable under acidic conditions. Have good dispersion and chemical stability.	Tumor inhibition rate was 82.6%, 23.7% higher than the PTX group. Reduced liver damage and nanotoxicity.	[22]
Glycyrrhetinic acid—oleanolic acid		-	Hydrogen bonding; hydrophobic interactions.	-	Tumor inhibition rate was Enhanced from 50.5% to 69.5%. Reliable biosafety.	[89]
Glycyrrhetinic acid-Liquidambaric acid		-	Hydrogen bonding; hydrophobic interactions.	-	Tumor inhibition rate was Enhanced from 37.3% to 82.9%. Reliable biosafety.	[89]
Ursolic acid -EGCG-aptamer		73.6%	Hydrophobic interactions; hydrogen bonding.	pH-responsive, released rapidly in acid conditions.	Synergistic anticancer effect. Enhanced tumor immune infiltration.	[90]

EGCG: epigallocatechin gallate.

## Data Availability

No new data were created or analyzed in this study. Data sharing is not applicable to this article.
